# Loss of TRPV4 reduces pancreatic cancer growth and metastasis

**DOI:** 10.1172/jci.insight.196280

**Published:** 2025-10-16

**Authors:** Joelle M.-J. Romac, Sandip M. Swain, Nidula Mullappilly, Bandana Bindhani, Rodger A. Liddle

**Affiliations:** 1 Department of Medicine and; 2 Department of Veterans Affairs, Duke University, Durham, North Carolina, USA.

**Keywords:** Gastroenterology, Oncology, Cancer, Fibrosis, Ion channels

## Abstract

Pancreatic ductal adenocarcinoma (PDAC) is a rapidly metastasizing cancer characterized by a dense desmoplastic stroma composed of extracellular matrix (ECM) proteins, which complicates treatment. Upon stimulation, pancreatic stellate cells (PSCs) differentiated into cancer-associated fibroblasts (CAFs) that are the source of ECM and cytokines in PDAC. We previously reported that mechanical stress activates PSCs and induces fibrosis through mechanical ion channel PIEZO1-mediated TRPV4 channel activation, but its role in PDAC remains unclear. Here we report that pathological activation of PIEZO1 differentiated human PSCs into an inflammatory CAF phenotype that expresses chemoresistance and cancer stemness markers CD10 and GPR77. In an orthotopic PDAC model, TRPV4-KO mice exhibited a significant reduction in tumor size, circulating inflammatory cytokines, tissue inhibitor of metalloproteinases-1 (TIMP1), and premetastatic niche markers, serum amyloid A (SAA) proteins. A similar trend was observed in mice lacking functional PIEZO1 in PSCs. The livers of TRPV4-KO mice exhibited fewer cancer cell microlesions, lacked macrotumors, produced lower levels of inflammatory protein S100A8, and developed fewer inflammatory cell clusters. In orthotopic and genetically engineered models of PDAC, these mice also had improved survival, suggesting that blocking TRPV4 channels may be a promising therapeutic target for PDAC.

## Abstract 2

<span>TRPV4 deletion in mice significantly reduced PDAC growth, metastasis and improved survival, suggesting that a </span><span>TRPV4 blocker could be beneficial in treating PDAC patients.</span>

## Graphical abstract

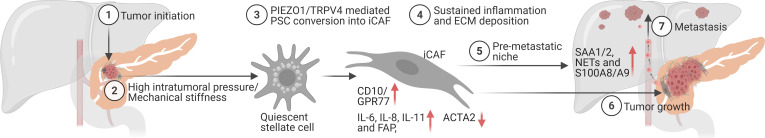

## Introduction

Pancreatic cancer is the third leading cause of cancer death in the United States, with an overall 5-year survival rate of 13% (1). It has become one of the deadliest cancers due to difficulty in early diagnosis and resistance to treatment. Pancreatic ductal adenocarcinoma (PDAC) is the most prevalent form of pancreatic cancer, comprising 90% of all pancreatic cancers (2). Currently, the lack of methods for early detection and the unique features of the tumor, characterized by a dense desmoplastic stroma, contribute to the poor outcome of this disease (3). This stroma, making up 80% of the tumor mass (4), consists of ECM proteins, such as fibronectin, collagen, hyaluronan, and laminin, produced by cancer-associated fibroblasts (CAFs), as well as infiltrating immune and endothelial cells (3, 5–8). Dense desmoplastic stroma acts as a barrier that collapses the microvasculature, thereby preventing chemotherapeutics from accessing tumor cells (6). Recently, distinct subtypes of CAFs have been identified within this stroma (9–13). Among these, α-smooth muscle actin–positive (α-SMA^+^) and fibroblast activation protein–positive (FAP^+^) CAFs, often referred to as myofibroblasts (myCAFs), are located near PDAC cells (11, 14) and may play a role in inhibiting tumor growth (15). In contrast, FAP^+^ CAFs that are situated farther from the tumor exhibit low levels of α-SMA and high levels of inflammatory cytokines such as IL-6, IL-11, and Leukemia inhibitory factor (LIF), categorizing them as inflammatory CAFs (iCAFs) (9–12, 14). Single-cell RNA-Seq has identified a third subtype known as antigen-presenting CAFs (apCAFs), which express CD74 and MHC class II antigens, hence displaying immune-modulatory functions; however, they are found in fewer numbers in human PDAC, and their precise role remains unclear (14). Furthermore, a subset of CAFs positive for the surface markers CD10 and GPR77 is associated with chemoresistance, cancer cell stemness, and poorer survival rates in solid tumors, including breast and lung cancers (16). In the context of PDAC, CD10^+^ PSCs significantly enhance the invasiveness of cancer cells, but the mechanistic role of CD10^+^GPR77^+^ CAF in pancreatic cancer progression and metastasis is unknown (16, 17).

Pancreatic cancer is known for its aggressive metastasis, with over half of patients presenting with metastases at the time of diagnosis, primarily affecting the liver (18, 19). This process is facilitated by the creation of a prometastatic environment, which allows circulating cancer cells to thrive (19). The iCAFs are derived from pancreatic stellate cells (PSCs), which account for 10% of total CAFs and produce high levels of IL-6 (12). iCAFS are the main cells expressing the cytokine in an organoid model of PDAC (20). IL-6 activates STAT3 signaling in hepatocytes, leading to the increased production of serum amyloid A1 and A2 (SAA1/2), proteins essential for the innate immune response (11, 12, 18, 21, 22). SAA1/2 expression is under the control of the inflammatory cytokines IL-6, IL-1β, and TNF (22). In PDAC, the persistent expression of SAA triggers chronic inflammation, promoting myeloid cell infiltration and aiding metastasis through the IL-6/STAT3/SAA signaling pathway (18).

Immune system imbalance contributes to PDAC progression (23, 24). CAFs promote an immunosuppressive microenvironment by induction and accumulation of M2-polarized tumor-associated macrophages (TAM), which secrete TNF-α, TGF-β, and IL-6, leading to PDAC progression and metastasis (8, 25, 26). CAF-derived IL-6 and GM-CSF cooperate to induce transdifferentiation of tumor-resident macrophages to M2 macrophages (8). Therefore, iCAFs are most likely responsible for inducing M2 polarization. In pancreatic cancer, overexpressed inflammatory proteins S100A8 and S100A9 are released by monocytes and neutrophils and attract inflammatory cells to the tumor site. This interaction stimulates tumor cells to produce proinflammatory cytokines, fueling further immune responses and promoting cancer cell invasion and migration (27–29). Additionally, the tissue inhibitor of metalloproteinase I (TIMP1) is elevated in many cancers, including PDAC, which correlates with poor prognosis (30, 31). TIMP1 exhibits multiple functions (32), and inhibition of metalloproteases was the first recorded function of TIMP1. TIMP1 also acts as a cytokine, influences the premetastatic niche by activating hepatic stellate cells, and induces the formation of neutrophil extracellular traps (NETs), which are composed of DNA fibers and proteins from neutrophils such as histone, myeloperoxidase (MPO), and elastase (33, 34). Those NETs trigger ECM remodeling and fibrosis within the tumor microenvironment (35).

PDAC is characterized by a tumor microenvironment that exhibits biomechanical alterations in which tumor cells initially increase intratumoral pressure (3, 5–8). Recent evidence indicates that PDAC cells can produce ECM along with CAFs, creating a mechanically stiff environment (3, 5, 36–38). Interstitial fluid pressure is significantly higher in Kras and p53 mutant tumors than in normal pancreatic tissue, emphasizing the roles of solid stress, fluid pressure, and microarchitecture in PDAC (6, 37). We previously reported that the pancreas is sensitive to pressure due to the expression of the calcium-permeable mechanically activated ion channel, PIEZO1 (39–41). Increased intraductal pressure activates PSCs via PIEZO1-mediated TRPV4 signaling, producing sustained calcium influx resulting in a fibrogenic response (40). This sensitivity of PSCs to pressure plays a critical role in the development of pancreatic fibrosis, emphasizing the involvement of PIEZO1 and TRPV4 in this pathological process (40). While much is known about the effects of mechanical stress on cancer cell behavior and PSCs, the specific roles of PIEZO1 and TRPV4 in PDAC remain unexplored. This study highlights the critical role of PIEZO1/TRPV4 in the differentiation of PSCs into an iCAF phenotype, which significantly influences PDAC growth and metastasis in orthotopic and genetically engineered mouse models of PDAC.

## Results

### Activation of PIEZO1 by Yoda1 in human stellate cells induces differentiation into a FAP^up^-iCAF phenotype.

**Figure 1:**
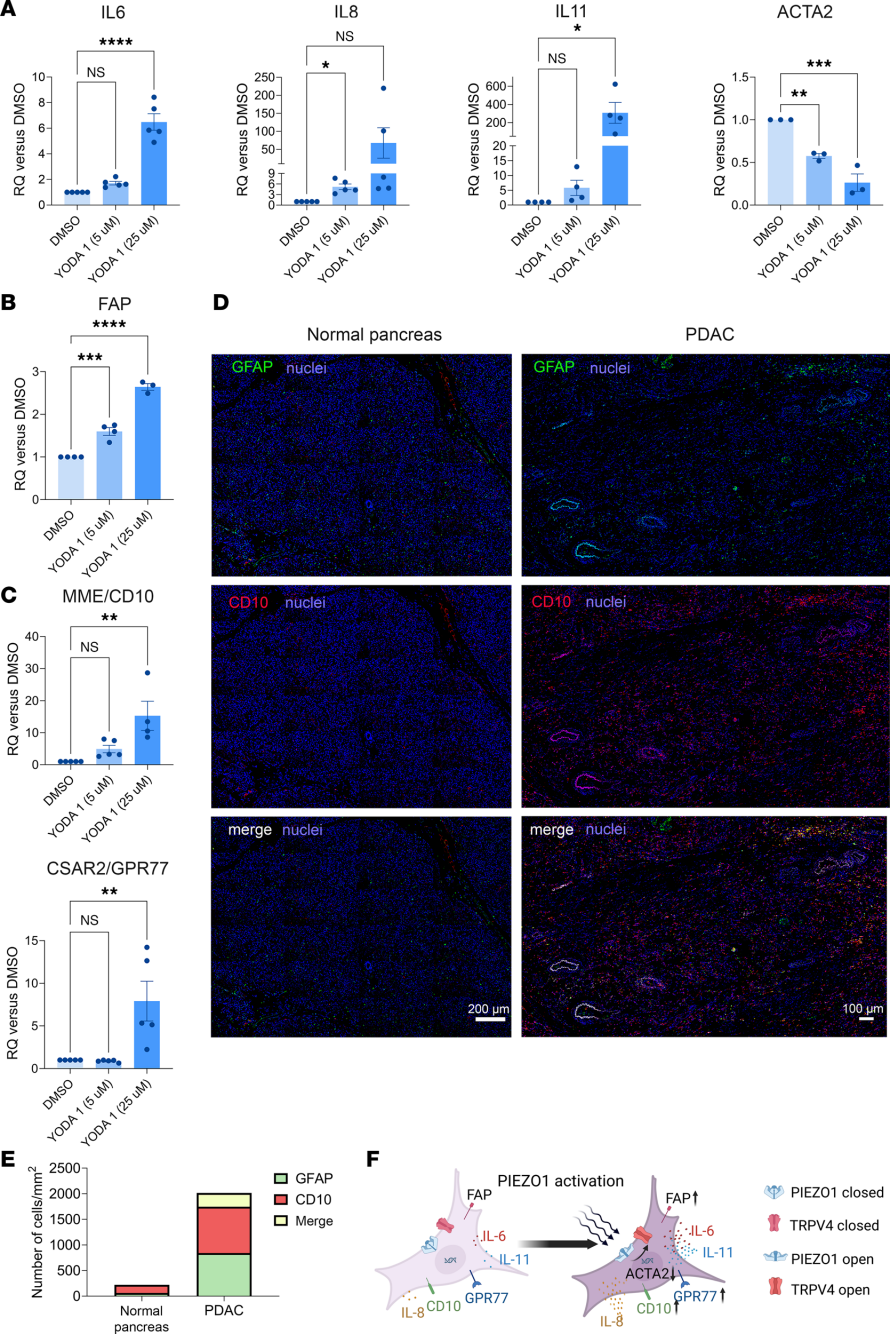
Piezo1 activation converts human PSCs to a FAP^up^-iCAF phenotype. Isolated human PSCs were incubated with Yoda 1 (5 or 25 μM) for 24 hours. (**A**–**C**) RNA was isolated, and RT-PCR assays were performed using primers for IL-6, IL-8, IL-11, and ACTA2 (**A**), FAP (**B**), and CD10 and GPR77 (**C**). Five human pancreas samples were used for the isolation of PSCs and in vitro studies. (**D**) Immunostaining of human normal pancreas or human PDAC with GFAP and CD10 antibodies. Note the colocalization of CD10 and GFAP. Scale bars: 200 μm (left) and 100 μm (right). (**E**) Stack bar representing the number of GFAP, CD10, and colocalized GFAP-CD10 cells in normal pancreas or PDAC tissue indicated that a similar number of CD10^+^ or GFAP^+^ were present in the PDAC tissue. No cell expression of both CD10^+^ and GFAP^+^ could be detected in the normal pancreatic tissue. (**F**) Graphical display recapitulating the phenotypic changes in human pancreatic stellate cells upon Piezo1 activation. Statistical analyses were performed using 1-way ANOVA with Dunnett’s post hoc test. Results are expressed as mean ± SEM. *P ≤ 0.05; **P ≤ 0.01: ***P ≤ 0.001; ****P ≤ 0.0001.

Human stellate cells are sensitive to mechanical activation through their expression of PIEZO1 (40). PIEZO1 is a mechano-gated ion channel that is primarily permeable to Ca^2+^ (42). Yoda1, identified as a specific agonist for PIEZO1 channels, was discovered following the screening of 3.25 million synthetic molecules (43). Here, we demonstrate that selective stimulation of PIEZO1 using the chemical agonist Yoda1 increased the expression levels of the cytokines IL-6, IL-8, and IL-11 while decreasing α-smooth muscle actin gene (ACTA2) expression; which is characteristic of iCAFs (Figure 1A) (11). Gene set enrichment analysis of RNA-Seq of Yoda1-treated murine stellate cells indicated that HALLMARK IL-6_JAK_STAT3_ signaling and HALLMARK inflammatory response were upregulated (Supplemental Figure 1; supplemental material available online with this article; https://doi.org/10.1172/jci.insight.196280DS1). In addition to these inflammatory mediators, expression of FAP, a marker of tumorigenicity in PDAC, was upregulated (Figure 1B) (44). In 2019, Elyada et al. classified inflammatory (iCAF), myofibroblast (myCAF), and antigen-presenting (apCAF) phenotypes (14). Recently, an in-depth study using single-cell RNA-Seq identified 6 clusters of CAFs based on genes commonly expressed in CAFs and genes specific to each subset (45). iCAF, myCAF, and apCAF were part of 3 nonoverlapping clusters. High expression levels of FAP were associated with poorer survival rates. In contrast, higher levels of SMA correlated with better survival outcomes (45). Moreover, in a Kras genetically engineered mouse model (GEMM), reducing FAP levels led to a significant decrease in tumor progression (46, 47).

We found that PIEZO1 activation increased expression of CD10 and GPR77 (Figure 1C). CD10^+^ and GPR77^+^ CAFs have been shown to promote cancer cell stemness and metastasis (16, 17). Interestingly, glial fibrillary acidic protein (GFAP), a marker for PSCs (40), and CD10 are coexpressed in the same cells throughout the stroma of human PDAC (Figure 1D and Supplemental Figures 2 and 3). Similar numbers of CD10^+^ or GFAP^+^ cells were present in the PDAC tissue (905 CD10^+^ and 842 GFAP^+^ cells per mm² area, respectively). Of those, 268 cells per mm^2^ area were both GFAP^+^ and CD10^+^. In contrast, we could not detect a cell expressing both CD10 and GFAP in normal pancreas. Overall, fewer CD10- or GFAP-expressing cells were present in the normal tissue (159 CD10^+^ or 63 GFAP^+^ cells per mm² area) (Figure 1E). We previously demonstrated that PIEZO1 and TRPV4 are expressed in human PSCs (40). In human PDAC, PIEZO1 and TRPV4 are also coexpressed in the same cells (Figure 1, A–F, and Supplemental Figure 4). Moreover, these cells exhibit high expression of markers associated with tumor progression (FAP) and cancer cell survival (CD10 and GPR77). Realizing that pathological PIEZO1 activation is linked to downstream activation of TRPV4 in chronic pancreatitis and inflammation (40), we next investigated the potential role of TRPV4 in PDAC.

### Loss of TRPV4 reduces tumor growth and metastatic niche formation.

**Figure 2:**
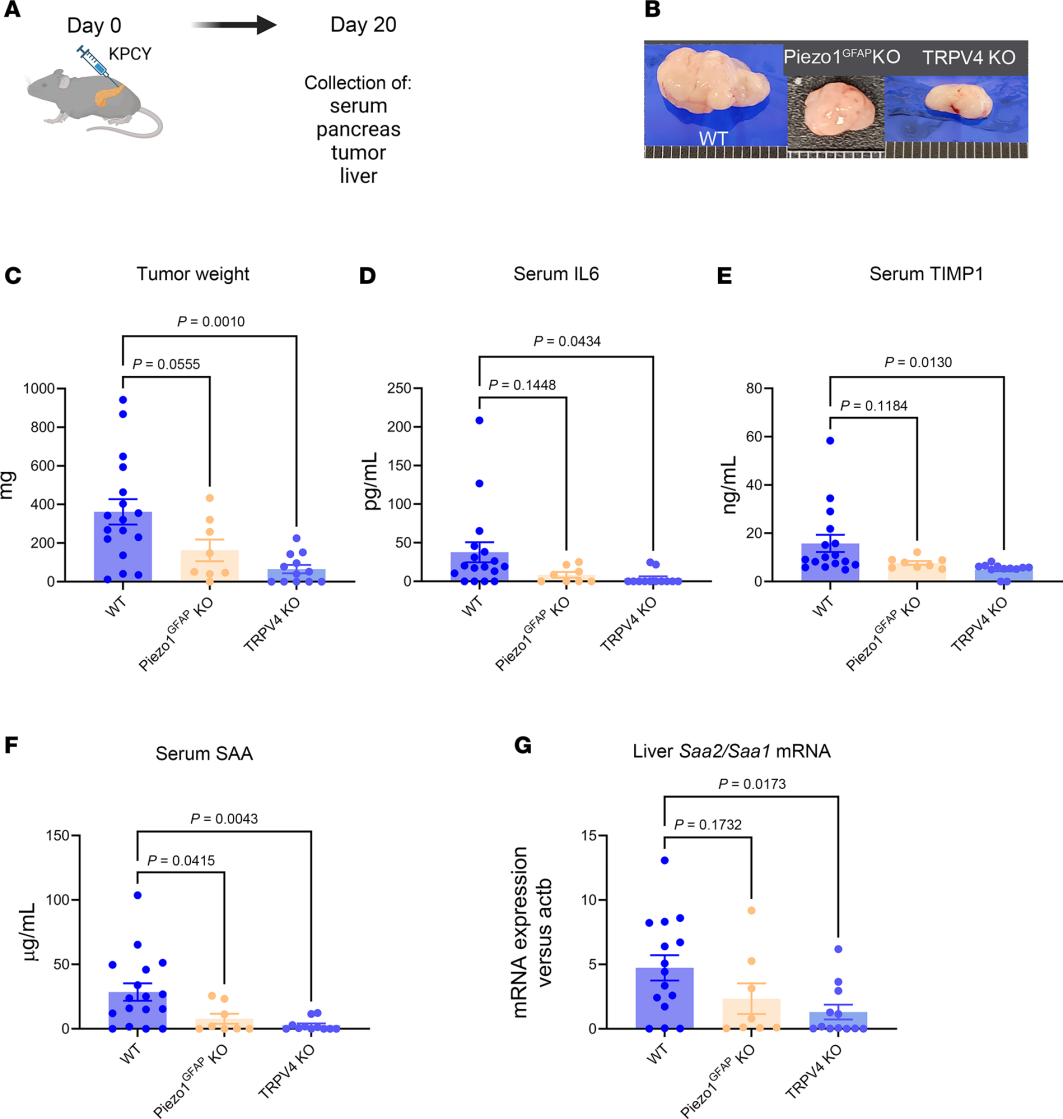
Loss of TRPV4 reduces tumor growth and cytokine expression known to activate hepatocytes and hepatic stellate cells. (**A**) Schematic of the orthotopic experiment. (**B**) Representative images of tumors from WT, Piezo1^GFAP^ KO, or TRPV4-KO mice that were injected with 100,000 KPCY cells in the tail of the pancreas. Organs were collected 20 days after surgery. (**C**) Quantitative analysis of tumor size. (**D**–**F**) Serum levels of IL-6 (**D**), TIMP1 (**E**), and SAA (**F**) were measured by ELISA. (**G**) *Saa2*/*Saa1* from liver RNA was measured by RT-PCR. Statistical analyses were performed using 1-way ANOVA with Dunnett’s post hoc test. Results are expressed as mean ± SEM.

We used an orthotopic model of PDAC to determine whether PIEZO1 or TRPV4 would influence tumor development and/or metastasis. We first used a genetically modified mouse line in which PIEZO1 was deleted in GFAP-expressing cells (Piezo1^GFAP^ KO), thereby eliminating PIEZO1 in PSCs (40). KPCY cells bearing mutations in Kras and P53 genes express the yellow fluorescent protein used as a tracer (48). KPCY cells were injected into the tail of the pancreas of WT, Piezo1^GFAP^ KO, or TRPV4-KO mice (Figure 2A). TRPV4 KO is a global KO; however, the KPCY cells carry functional TRPV4. Twenty days after surgery, pancreatic tumor weights were lower in TRPV4-KO mice, indicating that the absence of TRPV4 in the stroma substantially reduced tumor growth (Figure 2, B and C). Tumors from Piezo1^GFAP^ KO were reduced albeit not significantly. Serum levels of IL-6, a cytokine known to activate hepatocytes and trigger premetastatic niche formation (19), were significantly reduced in TRPV4-KO mice (Figure 2D). Circulating levels of TIMP1, which is also known to activate hepatic stellate cells and promote the premetastatic niche (33), were also significantly reduced in TRPV4-KO mice (Figure 2E).

**Figure 3:**
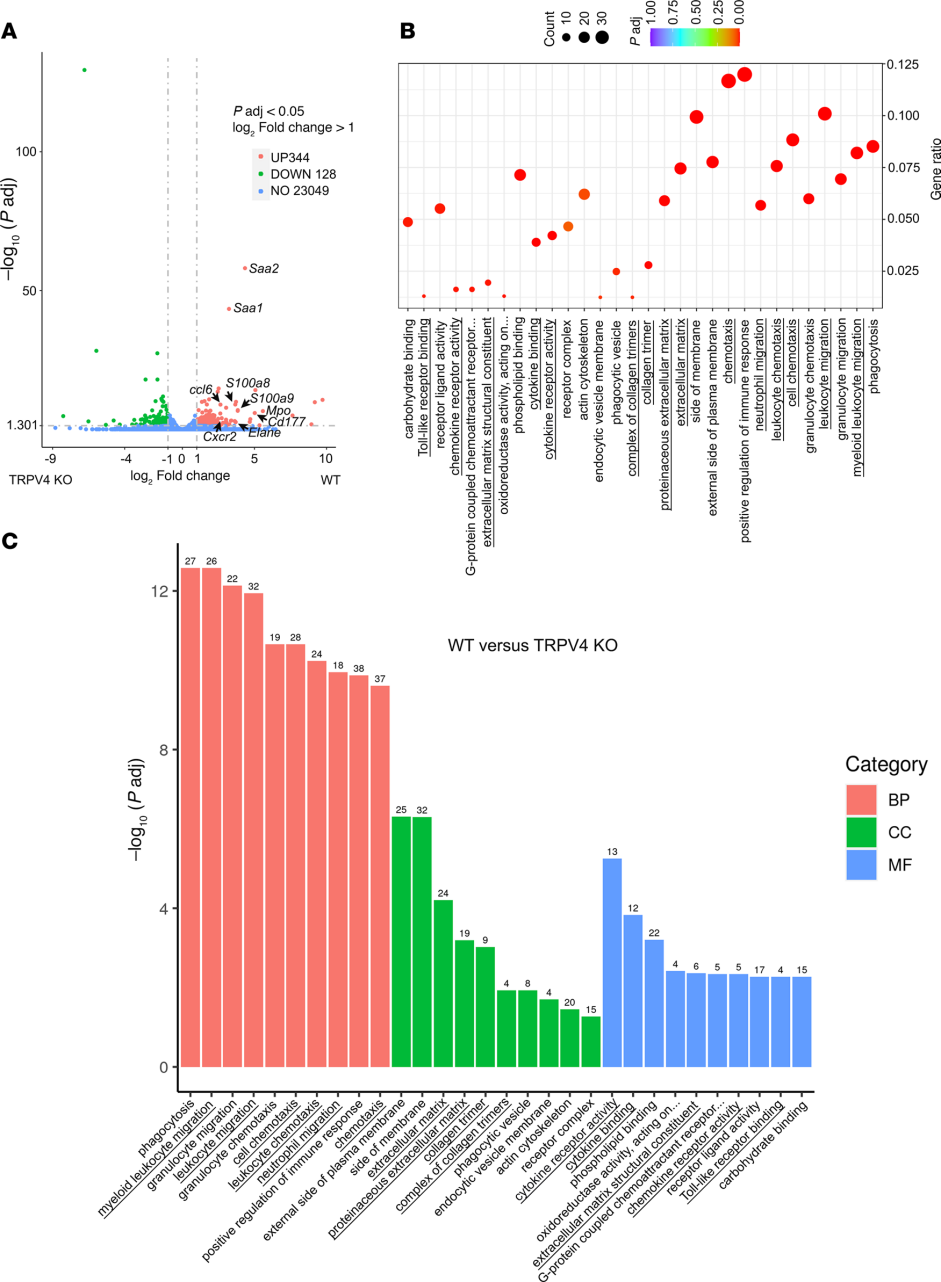
Reduction of innate immune response genes in an orthotopic model of PDAC in TRPV4-KO mice. Liver RNAs from WT or TRVP4-KO mice following orthotopic injection of KPCY cells (100,000 cells) were purified and analyzed by RNA-Seq. Upregulated WT genes versus TRPV4 KO; *n* = 2 animals for WT and *n* = 3 animals for TRPV4 KO. (**A** and **B**) Differentially expressed genes (DEGs list) volcano plot (**A**) and gene ontology (GO) scatter plot (**B**) listing the 10 highest upregulated pathways in WT versus TRPV4 KO: biological process (BP), cellular component (CC), and molecular function (MF). (**C**) Corresponding bar graph.

Previously, it has been demonstrated that IL-6 produced by pancreatic tumors induced hepatocyte *Saa1/Saa2* genes expression (19). SAA proteins are chemoattractants for myeloid cells, neutrophils, and monocytes and are considered markers for metastatic niche formation. Circulating levels of SAA proteins were significantly reduced in TRPV4-KO mice after orthotopic KCPY cell injection (Figure 2F) and correlated with a significant reduction in liver expression of *Saa1/Saa2* genes in TRPV4-KO mice (Figure 2G), indicating that hepatocytes were responsible for the increase in circulating SAA proteins. We observed a significant reduction in circulating SAA protein in Piezo1^GFAP^ KO mice. Expression of liver *Saa1/Saa2* genes was also reduced, albeit not significantly. Differentially expressed hepatic genes from WT versus TRPV4-KO mice were analyzed using RNA-Seq. A volcano plot (Figure 3A) depicted 344 genes that were more highly expressed in WT versus TRPV4-KO mice, including *Saa1*, *Saa2*, *S100a8*, *S100a9*, *Mpo*, *Elane*, *Cd177*, C*cl6*, and *Cxcr2*. Some of these genes are either selectively (e.g., *Elane*, *Cd177*) or predominantly (e.g., *Mpo*, *Cxcr2*) expressed in neutrophils (49–52). Notably, the expression of the *Elane* and *Mpo* genes, which indicate neutrophil infiltration, increased by 13-fold and 48-fold, respectively. *S100a8* and *S100a9* genes are highly expressed in neutrophils (53) and activated macrophages (54). *Ccl6* is expressed in myeloid cell lineages (55) and is involved in metastatic tumor growth (56). The chemoattractant proteins S100A8/A9 are involved in myeloid cell migration (57, 58), and the neutrophil-specific CD177 may facilitate transendothelial migration of neutrophils (59). CXCR2 contributes to metastatic niche formation (60), and neutrophil elastase is involved in ECM remodeling and awakening dormant cancer cells (61). The gene ontology (GO) enrichment scatter plot (Figure 3B) and bar graphs (Figure 3C) indicated that genes involved in migration and chemotaxis of cells from the innate immune system (leukocytes, neutrophils, granulocytes) were upregulated in WT animals (Figure 3C). In WT animals, there was a significant increase in cellular components associated with the ECM and enhanced cytokine-related functions, emphasizing the inflammation and extracellular remodeling in WT animals that promote metastasis (Figure 3C).

**Figure 4:**
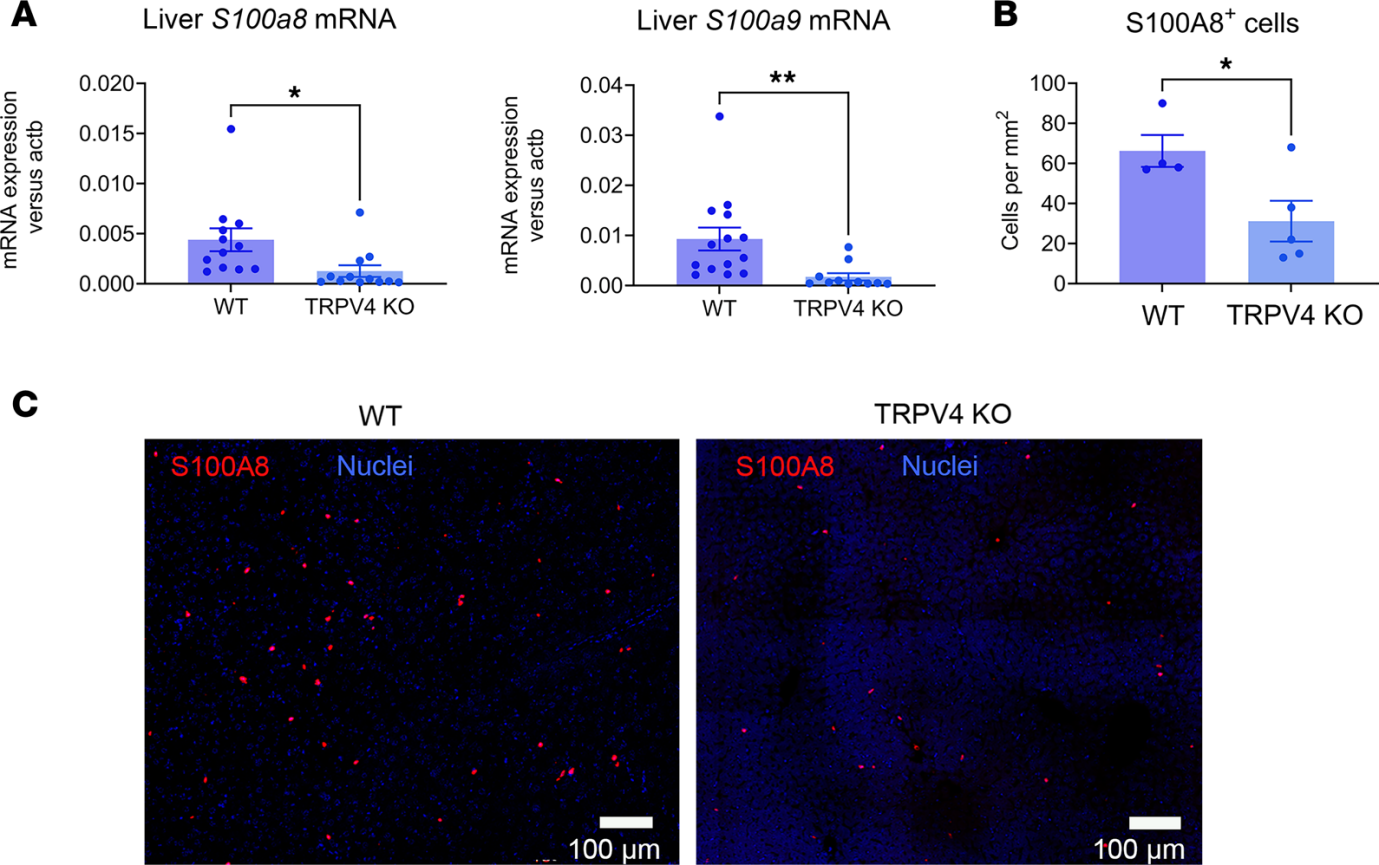
S100A8^+^ cells are reduced in livers of TRPV4-KO mice. (**A**) mRNA levels for *S100a8* and *S100a9* genes measured by RT-PCR. (**B**) S100A8^+^ cells were counted in liver sections of WT animals (*n* = 4) and TRPV4-KO animals (*n* = 5). (**C**) Representative image of immunostaining of S100A8 protein in liver sections of WT and TRPV4-KO mice. Statistical analysis was performed using Student’s *t* test. Results were expressed as mean ± SEM. Scale bars: 100 μm. *P ≤ 0.05; **P ≤ 0.01.

**Figure 5:**
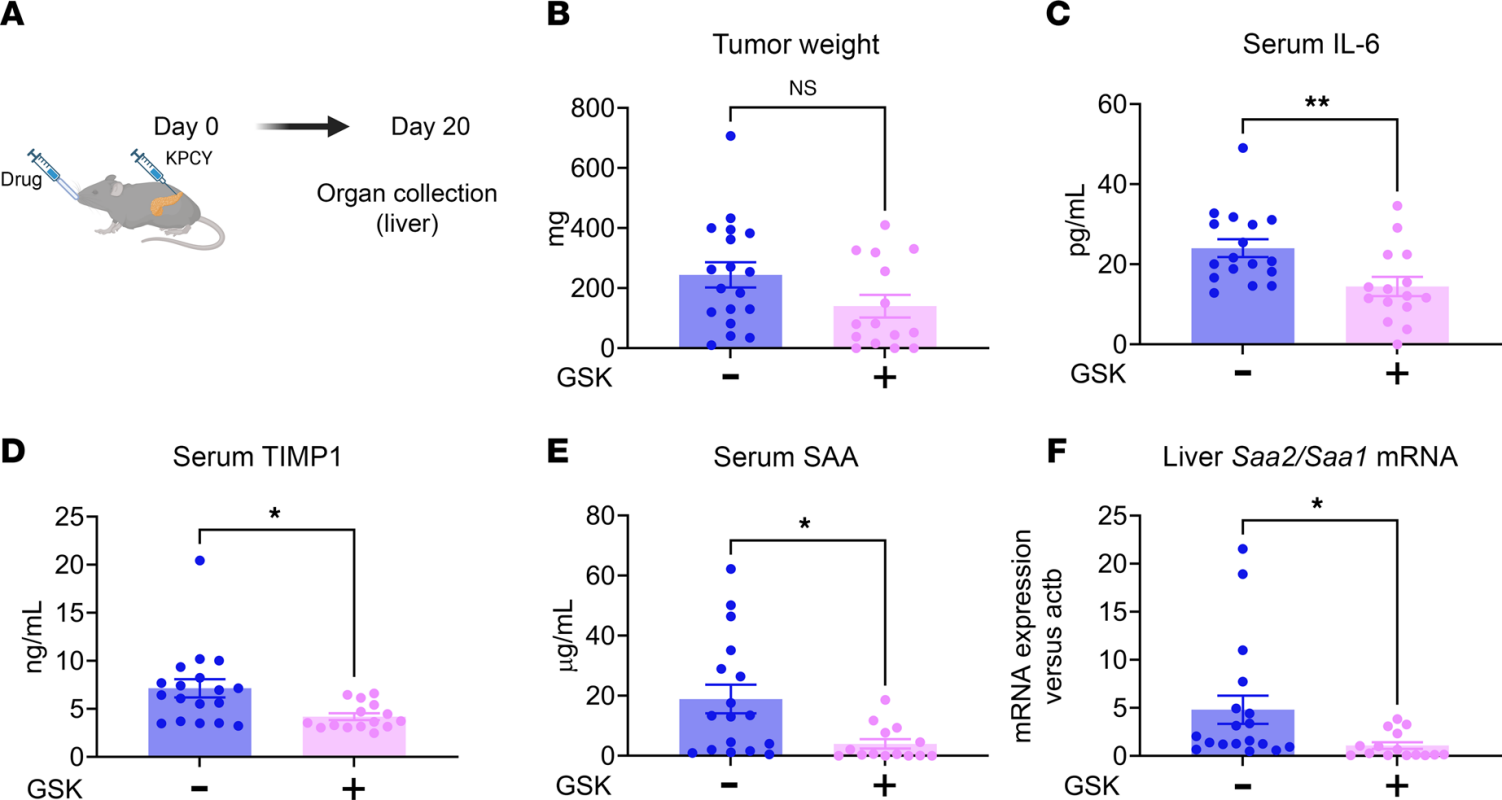
The TRPV4 antagonist GSK2193874 protects mice from premetastatic niche formation. (**A**) Graphical illustration of the orthotopic surgery with daily gavage of GSK2193874 (10 mg/kg). (**B**) Quantitative analysis of tumor weight. (**C** and **D**) Serum cytokines levels IL-6 (**C**) and TIMP1 (**D**) were measured by ELISA. (**E** and **F**) Serum SAA was measured by ELISA (**E**) and liver expression of *Saa1*/*Saa2* genes (**F**) by RT-PCR. Statistical analysis was performed using Student’s *t* test. Results were expressed as mean ± SEM. *P ≤ 0.05; **P ≤ 0.01.

**Figure 6:**
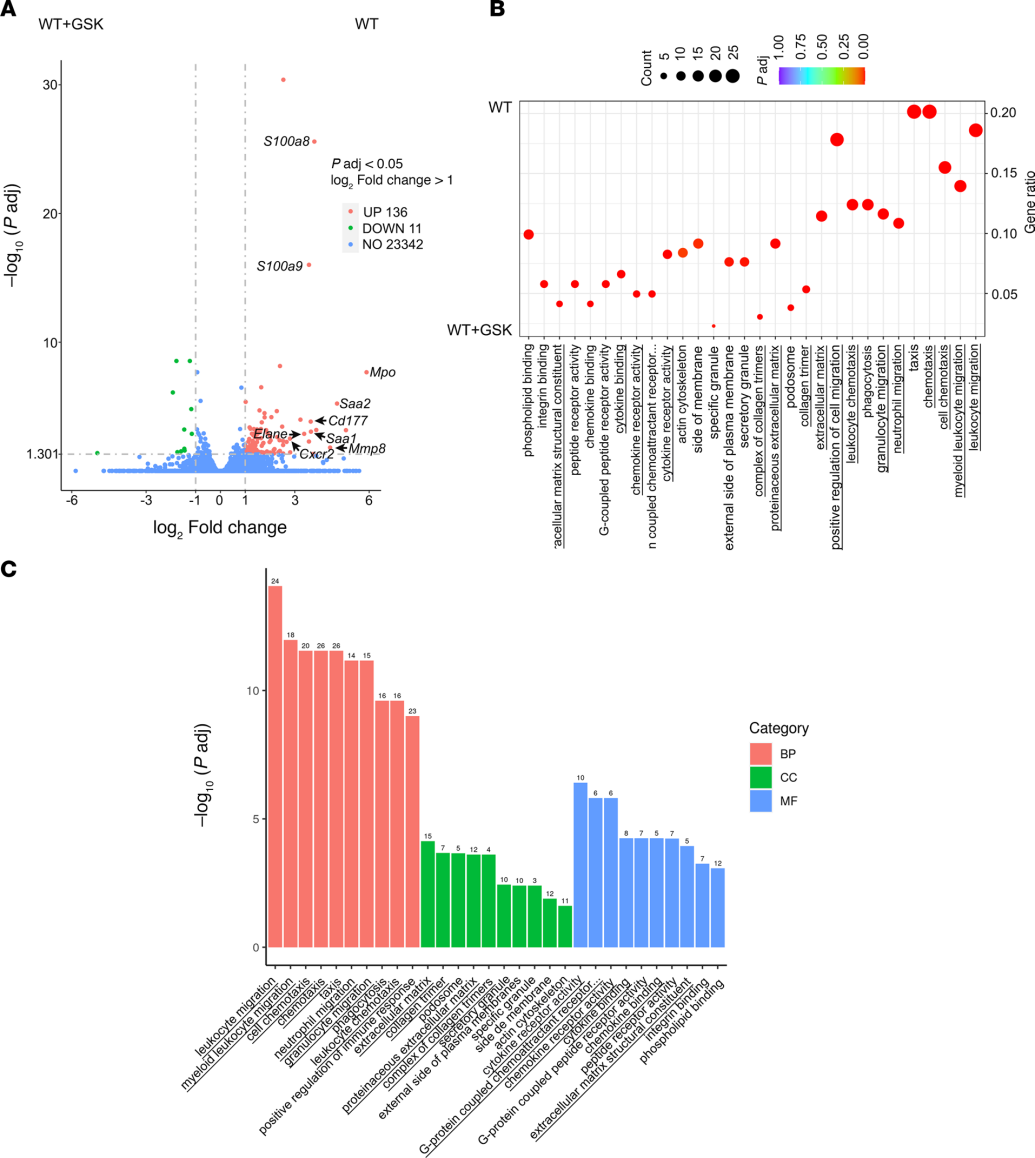
The TRPV4 antagonist GSK2193874 reduces the innate immune response in an orthotopic model of PDAC. (**A**) Differentially expressed genes (DEGs list) volcano plot from liver RNA-Seq analysis of WT mice and mice treated with GSK2193874 (10 mg/kg). (**B** and **C**) Gene ontology enrichment analysis showing scatter plot (**B**), and bar graph (**C**) of upregulated pathways.

Liver mRNA levels for the *S100a8* and *S100a9* genes were significantly higher in WT versus TRPV4-KO animals after orthotopic injection (Figure 4A). Immunofluorescence staining of liver sections also indicated that S100A8^+^ cells were more abundant in the WT animals versus TRPV4-KO animals (Figure 4, B and C). These results align with the data extracted from RNA-Seq, highlighting the significance of TRPV4 in the chemotaxis of innate immune cells to the liver. Based on the preceding experiments, it appeared that genetic deletion of TRPV4 was responsible for a reduction in tumor development and metastasis in an orthotopic model of PDAC. To determine whether pharmacological inhibition of TRPV4 would reduce the growth and spread of pancreatic cancer, we utilized a commercially available TRPV4 blocker (GSK2193874, referred to as GSK219) to assess its effects in an orthotopic model of PDAC. We treated mice with GSK219 for 20 days after orthotopic injection of KPCY cells (Figure 5). Circulating blood levels of IL-6 and TIMP1 were significantly reduced in treated animals (Figure 5, C and D). Levels of the metastatic niche formation markers SAA (Figure 5E) were also reduced and correlated with decreased hepatic gene expression (Figure 5F). From RNA-Seq data analysis, we noted that the same genes/pathways were affected in TRPV4 antagonist–treated WT animals and TRPV4-KO mice (Figure 3, A–C, and Figure 6, A–C).

### TRPV4 is necessary for liver metastasis.

**Figure 7:**
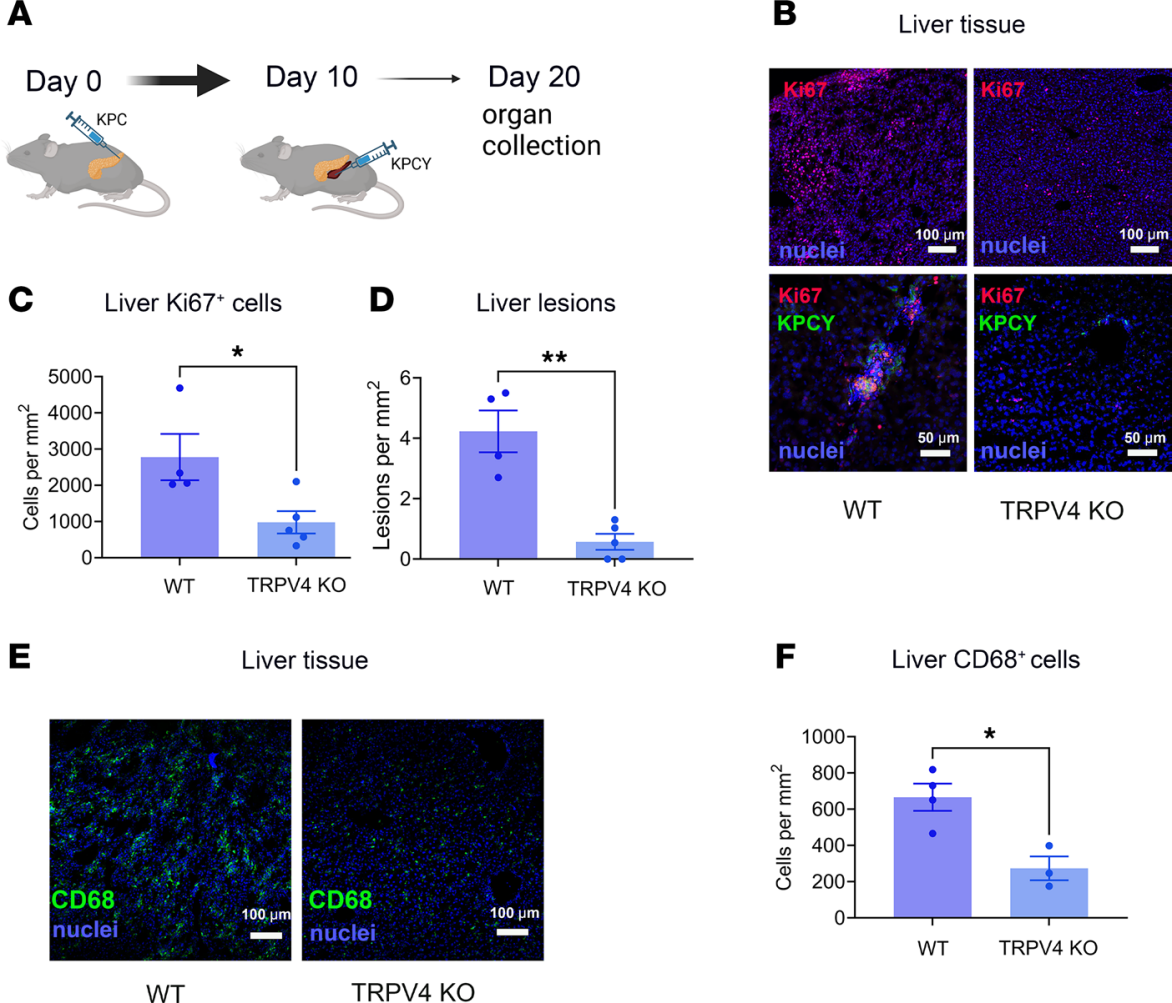
TRPV4 is necessary for hepatic metastasis of PDAC. (**A**) Graphical illustration of experimental design. KPC cells (100,000 cells) were injected into the pancreas, followed by injection of KPCY cells (500,000 cells) into the spleen 10 days later. (**B**) Representative images of immunostaining of mouse liver for Ki67 and GFP (marking KCPY cells) at low (upper panels) and high magnification (lower panels). Scale bars: 100 μm (top) and 50 μm (bottom). (**C** and **D**) Quantitative analysis of Ki67 positively stained cells and liver lesions of WT and TRPV4-KO liver. The analysis was performed on large sections of pancreatic tissues using tiling and stitching of images obtained with a Zeiss 880 Airyscan confocal microscope. Lesions were defined as an aggregate of at least 10 KPCY cells. (**E** and **F**) Representative images of immunostaining of liver sections with CD68 and quantitative analysis of CD68^+^ cells in liver sections from WT or TRPV4-KO mice. Scale bars: 100 μm. Statistical analyses were performed using Student’s *t* test. Animal number, *n* = 4–5. Results were expressed as mean ± SEM. *P ≤ 0.05; **P ≤ 0.01.

To determine if TRPV4 contributes to metastasis, we used an orthotopic model of pancreatic cancer metastasis with subsequent injection of cancer cells in the spleen (19). The second injection of cancer cells was necessary because metastasis from pancreatic tumors is an inefficient process (62, 63). KPC cells (100,000 cells) were first injected into the pancreas of WT or TRPV4-KO mice to induce the premetastatic niche; then, 10 days later, KPCY cells (500,000 cells) were injected into the spleen (Figure 7A). In the initial 10 days of the experiment, we expected that KPC cells would interact with their surrounding environment, develop into tumors, and initiate signals for metastatic niche formation in the liver through circulating factors released into the bloodstream. Consequently, by day 10, when KPCY cells were injected into the spleen, the liver would be primed for this metastatic process. As expected, using immunofluorescence staining with an antibody against the yellow fluorescent protein (specific for KPCY) and Ki-67 (nuclear staining in actively dividing cells), we found that WT livers harbored sites of PDAC cells (Figure 7). Clusters of KPCY cells were evident in the livers of WT mice, but very few KPCY cells were detectable in the livers of TRPV4-KO mice (Figure 7B). The rare KPCY cells that were visible appeared mostly as individual cells. Quantitative analysis of Ki67^+^ cells (Figure 7C) or lesions, defined as clusters of at least 10 KPCY cells, in the livers of WT or TRPV4-KO mice (Figure 7D) demonstrated that TRPV4 deletion protected mice from liver metastases. Additionally, CD68, which is highly expressed in macrophages, neutrophils, and other mononuclear phagocytes, was reduced in TRPV4-KO livers (Figure 7, E and F).

### TRPV4 deletion increases pancreatic cancer survival.

**Figure 8:**
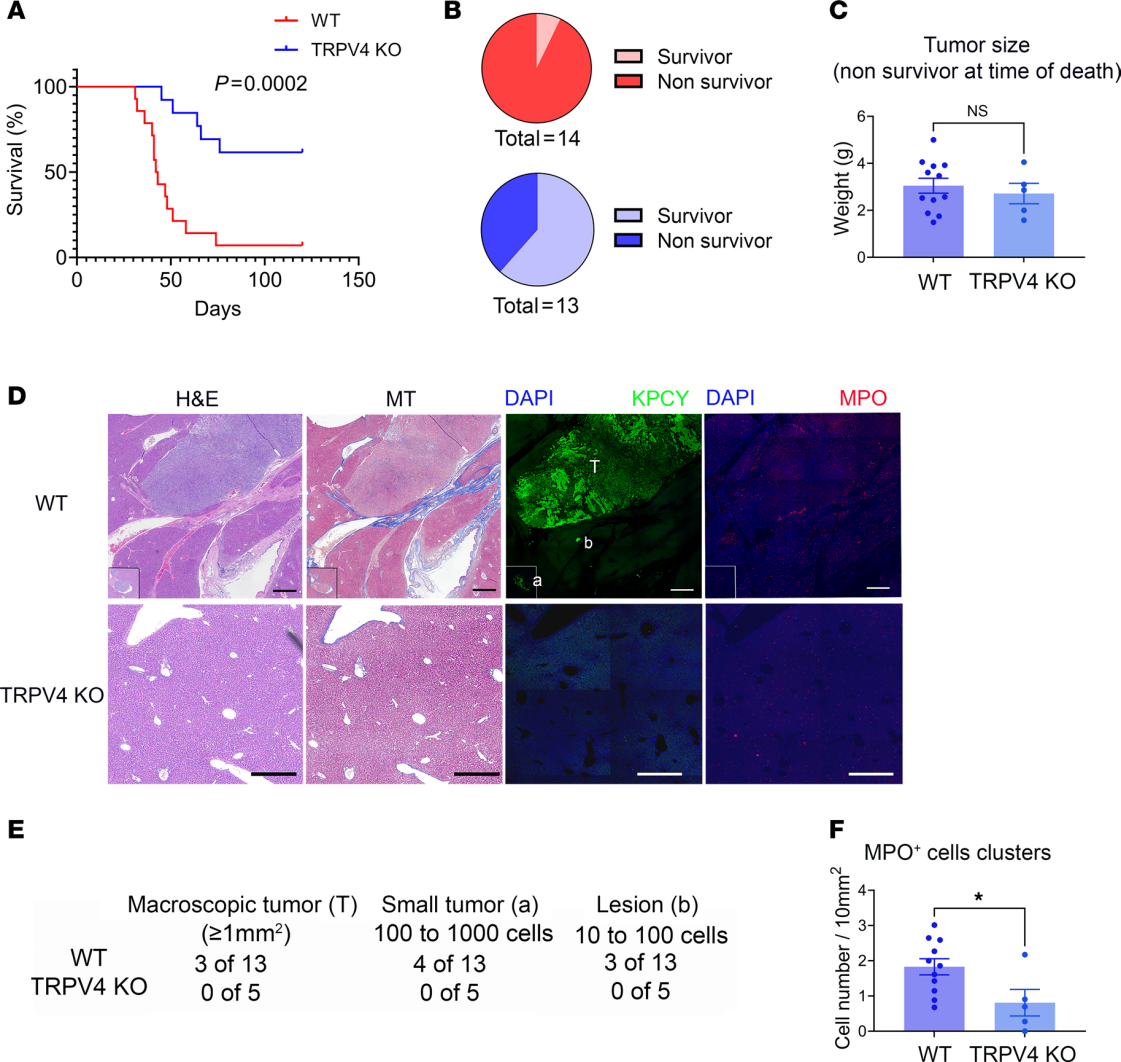
Effects of TRPV4 on pancreatic cancer survival. (**A**) Survival curve of WT and TRPV4-KO mice following orthotopic injection of KPCY cells (10,000 cells) into the pancreas. Mice were monitored 3 times per week for signs of distress. (**B**) Pie charts of survivors versus nonsurvivors at the end of the experiment (120 days) for WT (red) and TRPV4 KO (blue) mice. (**C**) Nonsurvivor mice pancreas tumor weight WT and TRPV4 KO at the time of their death. (**D**) Representative images of livers from WT mice with metastatic tumor (upper panels) and TRPV4-KO mice (lower panels). Scale bars: 1 mm. Serial sections were stained with H&E or Masson’s trichrome dye (left panels) and immunostained with GFP (marking KCPY cells) and MPO antibodies (right panels). (**E**) Computation of macroscopic tumor, small tumors, and tumor cell clusters from liver tissues of nonsurviving animals: *n* = 13 for WT and *n* = 5 for TRPV4 KO. (**F**) Quantitative analysis of MPO^+^ cell clusters (≥10 cells) from images of liver sections. MOP^+^ cells present in the tumors were excluded from the cell count. Comparison of survival curves was performed using the Log-Rank (Mantel-Cox) test. Statistical analysis was performed using Student’s *t* test. Results were expressed as mean ± SEM. *P ≤ 0.05.

We previously observed that TRPV4 deletion reduces tumor size and metastatic outcome within a defined time frame (20 days). To determine if TRPV4 deletion improved the survival of mice, we injected KPCY cells (10,000 cells) into the tail of the pancreas of WT or TRPV4-KO male and female mice. Mice were sacrificed when they exhibited obvious signs of distress (64, 65). As shown in Figure 8A, cancer survival was significantly greater in TRPV4-KO mice. More than half the TRPV4-KO mice were still alive at the end of the experiment (120 days) (Figure 8B). At the time of death, the tumor weights in WT and TRPV4-KO mice that required euthanasia were similar, averaging 3 grams (Figure 8C), and there was only a small amount of normal pancreatic tissue remaining. Unlike the primary tumors, the livers of TRPV4-KO mice differed significantly from their WT counterparts. Representative images of WT liver with a sizable metastatic tumor are shown in Figure 8D. H&E staining indicates a diseased area in WT animals and is characterized by extensive fibrosis, as demonstrated by Masson’s trichrome staining. KPCY cells express YFP-protein that cross-reacts with GFP antibody. GFP staining indicates the presence of numerous cancer cells in a liver section from a WT animal. Near the primary tumor, small tumors were seen embedded in the tissue (Figure 8D, Panel KPCY, lower left enlarged image insert). Of the WT mice that died during the experiment, 3 of 13 animals exhibited large liver tumors. Four mice had small tumors detectable on tissue sections, and 3 mice had lesions of 10–100 cells. In contrast, we were unable to detect any lesions, micro-, or macrotumors, in the liver sections of the 5 TRPV4-KO animals that died during the experiment (lower panel). None of the TRPV4-KO mice displayed metastatic tumors or macrolesions (Figure 8E). Immunostaining of neutrophils by MPO antibodies indicated that some neutrophil clusters were present in the livers of WT animals. Quantitative analyses of tiled images from the liver showed that MPO^+^ clusters were significantly more abundant in WT livers (Figure 8F).

### TRPV4 deletion increases pancreatic survival in a GEMM of PDAC.

**Figure 9:**
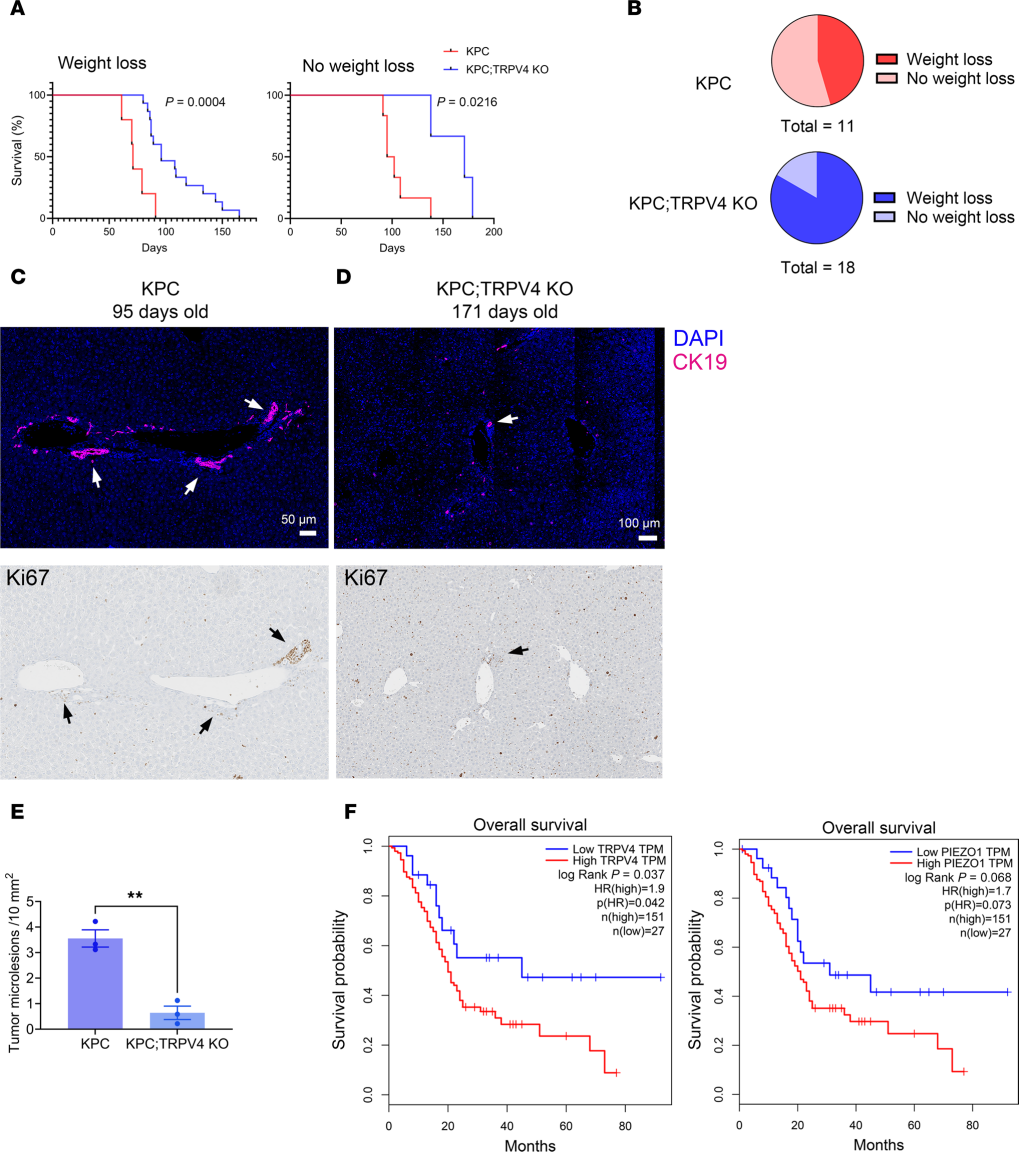
TRPV4 loss reduces metastases and increases survival in a GEMM model of PDAC and correlates with increased survival in human pancreatic ductal adenocarcinoma. (**A**) Survival curve of KPC versus KPC;TRPV4-KO mice. Mice either died with weight loss (left graph) or without weight loss (right graph). Log-rank values are indicated on the graph. (**B**) Distribution of animals in each category (loss of weight or absence of weight loss). (**C**) Representative images of liver tissues from KPC mice; upper panel: immunostaining with CK19 antibody; lower panel: IHC with Ki67. Sequential slides were used for staining. Scale bar: 50 μm. (**D**) Representative images of liver tissues from KPC;TRPV4-KO mice; upper panel: immunostaining with CK19 antibody; lower panel: IHC with Ki67. Sequential slides were used for the staining. Age (days) of each mouse at time of death is listed. Arrows indicate the presence of microlesions. Scale bar: 100 μm. (**E**) Quantitative analysis of tumor microlesions in liver tissues from KPC or KPC;TRPV4-KO mice. (**F**) Human pancreatic adenocarcinoma. Left panel: overall survival curves for patients with high TRPV4 expression (upper fifteenth percentile) versus patients with low TRPV4 expression (lowest fifteenth percentile). Right panel: overall survival curves for patients with high PIEZO1 expression (upper fifteenth percentile) versus patients with low PIEZO1 expression (lowest fifteenth percentile). Comparison of survival curves was performed using the Log-Rank (Mantel-Cox) test. Statistical analysis was performed using Student’s *t* test. Results were expressed as mean ± SEM; **P ≤ 0.01.

In the KPC mouse model of PDAC, we observed 2 distinct liver phenotypes. One group, which had to be euthanized due to significant weight loss as a humane endpoint, exhibited obstructive jaundice (66). The other group did not experience significant weight loss but was euthanized due to severe distress. Therefore, we studied the mouse survival rate in both these groups separately (Figure 9, A and B). Overall, the primary tumor sizes at the time of death were similar between the KPC and KPC;TRPV4-KO mice (Supplemental Figure 5). The group of mice characterized by weight loss showed considerable liver necrosis. The differences in phenotypes (weight loss versus no weight loss) may be attributed to the tumors’ locations; tumors near the common bile duct can cause obstructive jaundice, while those in the tail region may not. The liver necrosis we found resembled that observed after bile duct ligation (67). The presence of necrosis impaired the analysis of metastases in the liver of mice that had lost weight; thus, we focused on the second category of animals. Hepatic metastatic microlesions were identified through immunostaining with CK19 and the presence of dividing cells, indicated by Ki67, as shown in Figure 9, C and D. Overall, liver tissue from KPC mice exhibited a significantly higher number of microlesions compared with the mice lacking TRPV4 (Figure 9E). Notably, KPC;TRPV4-KO mice demonstrated longer survival than WT KPC mice in both groups.

Interestingly, TRPV4 and PIEZO1 expression levels in humans appear to influence the survival rates of patients with PDAC. Using the human Gene Expression Profiling Interactive Analysis (GEPIA) database (68, 69), we examined the expression of TRPV4 and PIEZO1 in patients with pancreatic adenocarcinoma (PAAD) and life expectancy. Our analysis revealed that patients with high tumor levels of TRPV4 (upper fifteenth percentile) had a significantly lower life expectancy compared with those in the lowest fifteenth percentile. Similarly, patients with high tumor levels of PIEZO1 expression (upper fifteenth percentile) also experienced lower life expectancy than those with low tumor PIEZO1 expression (lower fifteenth percentile) (Figure 9F).

## Discussion

In PDAC, a stiff extracellular matrix (ECM) characterized by dense stromal tissue significantly contributes to cancer progression. This stiffness triggers mechanotransduction pathways in cancer cells and adjacent cell types that may promote carcinogenicity. This study elucidates the roles of the mechanosensing ion channels PIEZO1 and TRPV4 in pancreatic tumor development and metastasis. PIEZO1 is upregulated in cancer cell lines and tumors of various cancers, including solid tumors such as breast cancer and glioma (70). Hallmarks of cancer that are modulated by PIEZO1 comprise sustained proliferative signaling, evasion of growth suppressors, sustained angiogenesis, immune evasion, and metastasis (71). Here we demonstrate that PIEZO1 is involved in the phenotypic conversion of PSCs into iCAFs. In the tumor microenvironment, CAFs sustain and enhance pancreatic tumor growth and metastasis (72). Our findings support previous studies that part of the CAF population originates from PSCs (12). In PSCs, high prolonged external force induces a fibrotic response through sequential activation of PIEZO1 and TRPV4 channels, and inhibition of either channel reduces fibrosis (40). We hypothesized that stiffness of the tumor environment may stimulate PIEZO1 in PSCs and trigger cellular differentiation (40, 73). Thus, PIEZO1 converted isolated quiescent human PSCs into an iCAF-like phenotype characterized by increased expression of cytokines (IL-6, IL-8, IL-11) and away from a myCAF phenotype, which is marked by decreased ACTA2 expression. We refer to these as pressure-induced iCAFs. iCAFs with these characteristics have been shown to be protumorigenic (11). Some of these iCAFs also expressed surface protein CD10: an endopeptidase involved in ECM remodeling and cancer cell invasiveness (17). In addition, a subset of CD10^+^GPR77^+^ CAFs have been shown to promote chemoresistance of breast cancer cells in patients (16). GPR77 is a membrane bound receptor for the complement system protein C5a. In breast cancer, binding of C5a to its receptor activates NF-κB, resulting in increased expression of the cytokines IL-6 and IL-8 (16). NF-κB is also a critical factor in pancreatic cancer proliferation, angiogenesis, and metastasis (74). Interestingly, we observed that the PIEZO1 agonist Yoda1 increased expression of CD10, GPR77, IL-6, and IL-8 in human PSCs, suggesting a possible protumorigenic and prometastatic contribution.

Having established that PIEZO1 activation in PSCs induced an iCAF phenotype, we were interested in determining if interrupting PIEZO1 signaling would affect PDAC development. The interplay between PIEZO1 and TRPV4 in pancreatic fibrosis and inflammation suggests that TRPV4 inhibition blocks the pathological responses triggered by PIEZO1 activation (40). Therefore, we used TRPV4-KO mice to assess PDAC progression in an orthotopic model of PDAC (19). We initially performed orthotopic experiments in mice lacking PIEZO1 in PSCs (Figure 2). We observed that tumor size, circulating cytokines IL-6 and TIMP1, and hepatic expression of *Saa1*/*Saa2* genes while statistically not different from WT animals trended lower; serum level of SAA protein was significantly lower. In TRPV4-KO mice, all those parameters were significantly reduced. The cytokine IL-6 is involved in premetastatic niche formation through activation of hepatocytes, which are the main source of SAA proteins and are responsible for the increased expression of SAA proteins in PDAC (19). SAA proteins exhibit immunosuppressive characteristics (75), which are integral to human PDAC (76). Those proteins are chemoattractant and are responsible for the inflammatory process occurring during premetastatic niche formation (77). Activated neutrophils play a crucial role in this process (76, 78). Thus, higher levels of chemoattractant proteins (SAAs, S100A8/A9) are a characteristic feature in the development of metastasis. In our experiments, those circulating proteins were reduced in the serum of TRPV4-KO mice, likely reducing innate immune cell migration. Consistent with this observation, we found decreased hepatic neutrophil and myeloid cell/macrophage infiltration in TRPV4-KO mice as indicated by differential expression of the genes: *S100a8/a9* (79), *Cd177* (52)*, Mmp8* (80)*, Mpo* (50)*, Elane* (49), and *Cxcr2* (60, 81, 82). In addition, TIMP1, which contributes to premetastatic niche formation by promoting inflammation (32–34), was decreased in the serum of TRPV4-KO mice. Chemical blockade of TRPV4 by the antagonist GSK2193874 yielded similar results on premetastatic niche formation (serum level of IL-6, TIMP1, and SAA proteins) to those observed in genetically modified mice. Tumor size tended to be smaller in mice treated with GSK2193874 (*P* = 0.0807). The reduced antitumor effect observed with the TRPV4 antagonist could be due to either the limited potency of GSK2193874 on TRPV4 channels or a dose effect. Additional studies with higher doses of drug could help clarify this issue.

The reduced premetastatic niche in TRPV4-KO mice was reflected by a reduction in hepatic metastases. In TRPV4-KO mice, every cell type except for the transplanted KPCY cells carries the *Trpv4* gene mutation; therefore, TRPV4 could affect tumorigenesis and metastasis through several interacting mechanisms and cell types. Both PIEZO1 and TRPV4 are expressed in numerous cell types including neutrophils and macrophages. TRPV4 is involved in neutrophil activation and induces reactive oxygen species (ROS) production, leading to inflammation (83). It is also present in macrophages, and its activation by mechanical cues is correlated with a phenotypic switch to M1 type macrophages characterized by proinflammatory cytokine expression and ROS formation (84, 85). Neutrophils are involved in cancer development and metastasis (86), and PIEZO1 activation in those cells contributes to NET formation and inflammation (87), a characteristic of PDAC. PIEZO1 induces the differentiation of monocytes into TAM through Protein Tyrosine Kinase 2 (PYK2) in the tumor microenvironment of PDAC. Those macrophages contribute to ECM remodeling and immune evasion, resulting in tumor growth. Genetic targeting deletion of the PYK2 in monocytes increases the effectiveness of immunotherapy (88). PIEZO1-TRPV4 interplay could also be an essential mechanism in immune cells involved in tumor progression and metastasis. We have demonstrated that TRPV4 is an essential effector for the PIEZO1 channel signaling in fibrosis (40). Additionally, the PIEZO1-mediated TRPV4 channel opening in pancreatic acinar cells triggers pancreatitis and produces loss of endothelial monolayer integrity under high-pressure situations (41, 89). In our experiments, in which PIEZO1 was only targeting PSCs, functional PIEZO1 in other cell types would still induce responses to mechanical cues and activate TRPV4. On the contrary, a global TRPV4 KO would shut off protumorigenic mechanisms in multiple cell types. Thus, the absence of PIEZO1 solely in PSCs was less effective at reducing tumor growth and premetastatic markers compared with mice with global deletion of TRPV4 KO. In the orthotopic transplant model, WT mice, but not TRPV4-KO mice, developed liver tumors. TRPV4-KO mice lived longer, and more than 50% of the TRPV4-KO mice were still alive at the end of the experiment. To confirm the survival advantages of TRPV4 deletion, we crossed KPC GEMM mice with TRPV4-KO mice to generate a PDAC model lacking TRPV4. Importantly, KPC;TRPV4-KO mice showed a significant survival advantage indicating a critical role of TRPV4 in PDAC development and progression. Both orthotopic and GEMM models of PDAC with loss of TRPV4 function showed a significant reduction in the number of metastatic microlesions in liver tissue. We did not observe any macrotumors in the livers of our KPC mice. At the time of reaching humane endpoints, both WT and KPC;TRPV4-KO mice had large pancreatic tumors with nearly complete loss of normal parenchyma.

In summary, we observed that pathological activation of PIEZO1 upregulated genes involved in tumor growth and metastasis in human PSCs. We also found that inhibiting downstream PIEZO1 signaling through TRPV4 could slow the progression of PDAC and reduce liver metastases. Interestingly, patients with PAAD exhibiting higher expression of PIEZO1 or TRPV4 had worse outcomes than those with low expression of either of these 2 genes, suggesting a potential role of PIEZO1 signaling in the emergence and progression of PDAC. Importantly, in our mouse models, both tumor size and metastasis could be reduced by eliminating or blocking TRPV4. These findings indicate that a TRPV4 blocker could be beneficial in treating patients with PDAC. Since metastasis is the leading cause of death in PDAC, targeting premetastatic niche formation and reducing metastatic tumors through TRPV4 inhibition may improve patient outcomes. Based on our findings from orthotopic and GEMM mouse models of PDAC, we propose that targeting TRPV4 may be a promising therapeutic strategy to enhance the lifespan of patients with pancreatic cancer.

## Methods

### Sex as a biological variable.

Our study included both male and female mice. No sex-specific differences were observed.

### Animals.

*Piezo^fl/fl^* mice were a gift of Ardem Patapoutian (Scripps Research Institute, La Jolla, CA) and were referred to as WT mice. *B6.Cg-Tg(GFAP-Cre/ERT2)* *Piezo1^fl/fl^* mice were generated as described (40) and are referred to as Piezo1^GFAP^ KO mice following treatment with tamoxifen. Mice with the *Trpv4^–/–^* mutation were a generous gift of Wolfgang Liedtke, Duke University, Durham, NC (90). KPC tamoxifen-inducible (Kras^LSL–G12D^ p53^LoxP^ Pdx1-CreER) mice were purchased from Jackson Labs (catalog 032429). They were crossed with *Trpv4^–/–^* to obtain the KPC;TRPV4-KO mice. Animals with Kras^het^ and p53^–/–^ were used in our experiments. Mice aged 8 to 12 weeks were used in all experiments except for the orthotopic or genetically engineered mouse PDAC survival studies, which included mice aged 3 to 6 months due to their varied humane endpoints. Within each experiment, animals were matched by sex and age to ensure consistency. Mice were housed under standard 12-hour light/12-hour dark conditions. Studies were approved by the Institutional Animal Care and Use Committee of Duke University.

### Materials.

Yoda1 (catalog 5586) was purchased from Tocris and resuspended at a concentration of 20 mM in DMSO. GSK2193874 was purchased from Millipore Sigma and was resuspended in 6% cavitron W7 HP7 (Ashland; catalog 826765) at 10 mg/kg and administered by orogastric gavage (250 μL) every 24 hours. Tamoxifen was purchased from Sigma (catalog T5648). Tamoxifen (80 mg/kg) was administered by IP injection (in 100 μL corn oil) to adult GFAP-Cre/ERT2;Piezo1^fl/fl^ mice for 5 consecutive days. Tamoxifen (6 mg in 250 μL corn oil) was administered to induce cre-expression in KPC or KPC;TRPV4-KO mice by oral gavage of the mother on postnatal days 0, 1, 2, and 4 (91).

### Antibodies.

The following antibodies from Abcam were used for immunostaining: Ki-67 antibody (ab16667), GFP antibodies (ab13970 or ab290), GFAP antibody (ab4674), CD10 antibody (ab256494), S100A8 antibody (ab92331), CD68 antibody (ab303565), MPO antibody (ab300650) and CK19 antibody (ab52625). TRPV4-ATTO-fluor-550 antibody was purchased from Alomone (ACC-034-AO) and PIEZO1 antibody from Proteintech (15939-1-AP).

### Cell lines.

KPCY cells were purchased from Kerafast (catalog EUP012_FP) (48). KPC cells were purchased from Cancertools (catalog 153474).

### Surgical procedures.

Orthotopic injections were performed following the protocol described (92) by injecting 100,000 cells (KPCY or KPC) suspended in phosphate-buffered saline (PBS) containing 1% Matrigel (Corning) in a volume of 20 μL using a 30 gauge needle in the tail of the pancreas. Animals were sacrificed 20 days after surgery, and pancreas and liver tissue were collected. Spleen injections of KPCY cells (500,000 cells in 50 μL PBS) were injected into the spleen following a surgical protocol similar to the orthotopic injection. The spleen injections occurred 10 days after the orthotopic injections. In this case, the cells used for orthotopic injections were the nonfluorescent KPC cells.

### In vitro cell studies.

Pancreatic tissues for human stellate cell preparation were provided by Duke University’s BioRepository & Precision Pathology Center under IRB approval. Human PSCs were isolated using a previously described protocol (40). The cells were isolated using collagenase digestion and cultured on a Matrigel-coated plate, supplemented with DMEM/F12 medium containing 10% fetal bovine serum (FBS). Cells were monitored for purity by immunostaining with GFAP antibody and for quiescent phase status by staining lipid fat droplets with Bodipy (40). Cells were used in experiments at passage 3 which yielded enough cells for experiments. PIEZO1 was activated using the PIEZO1 agonist Yoda 1. RNAs were isolated 24 hours later after Yoda1 treatment. Murine stellate cells were prepared as previously described (40) and cultured on a Matrigel-coated plate supplied with DMEM/F12 media with 5% fetal bovine serum (FBS) for 3 days. RNAs were isolated 24 hours after Yoda1 treatment using the Ribopure kit (Invitrogen, catalog AM1924). cDNAs were synthesized using the High-Capacity Reverse Transcription kit (Applied Biosystem, catalog 4368814).

### Assays.

RNAs for RNA-Seq analysis were performed by either the Duke Center for Genomic and Computational Biology core facility (human PSCs) or Novogene (mouse liver RNAs). RT-PCR was performed using the TaqMan assay (catalog 4331182) purchased from Life Technologies for IL-6 (Hs00174131_m1), IL-8 (Hs00174103_m1), IL-11 (Hs01055414_m1), ACTA2 (Hs01099348_m1), FAP (Hs00990791_m1), MME (Hs00153510_m1, CSAR2 (Hs01933768_s1), *Saa1*/*Saa2* (Mm04208126_mH), *S100a8* (Mm00496696_g1), and *S100a9* (Mm00656925_m1). Blood serum was collected by cardiac puncture, and ELISA for IL-6 was performed using the Mouse IL-6 Quantikine ELISA Kit (catalog M6000B) from R&D Systems following the manufacturer’s instructions. ELISAs were performed using the Abcam ELISA kits ab215090 and ab196265 for SAA and TIMP1, respectively.

### Immunostaining and microscopy.

Human normal pancreas and PDAC tissue sections of formalin-fixed and paraffin were provided by the Duke BioRepository & Precision Pathology Center. Antigen retrieval was performed following the manufacturer’s (Aptium Biologics) instructions using the Retriever 2100. All slides were incubated with the antigen retrieval buffer R-BUFFER A, pH 6 (Electron Microscopy Sciences; catalog 62706-10). Primary antibody dilutions were used according to the manufacturer’s recommendations and were incubated overnight at 4°C. Images were captured on a Zeiss 880 Airyscan fast inverted confocal microscope using a 20× objective with the tiling and stitching features. Murine paraffin-embedded formalin-fixed livers were sectioned at 5 μm. Serial sections were used for H&E staining, Masson’s trichrome staining, and immunostaining with S100A8, GFP, MPO, Ki67, and CK19 antibodies. Slides used for immunostaining were treated with TrueBlack Lipofuscin autofluorescence quencher (Cell Signaling Technology; catalog 92401). Primary antibody dilutions were used according to the manufacturer’s recommendations. Alexa Fluor 647– or Alexa Fluor 488–conjugated secondary antibodies used to visualize the immunostaining. The immunostaining images were obtained using a Zeiss Axio Observer wide-field microscope with a 10× objective. Images of a large section of the liver tissue were tiled and stitched. Ki67 (catalog ab16667) staining of GEMM mice liver were performed from paraffin embedded liver tissues by IHC at the Duke Biorepository and Precision Pathology center using the Ultra Discovery automated platform. Ki67 staining, H&E, and Masson’s Trichrome images were scanned at the Duke Biorepository and Precision Pathology Center using a Leica Aperio GT 450 scanner with a 40x objective. Paraformaldehyde-fixed frozen sections of murine liver were used for immunostaining with Ki67, CD68, and GFP. Alexa Fluor 647– or Alexa Fluor 488–conjugated secondary antibodies used to visualize the immunostaining. Images were captured using a Zeiss 880 Airyscan Fast Inverted Confocal microscope with a 20x objective and employing tiling and stitching features.

### Image analysis.

Quantification analysis of CD10^+^ and GFAP^+^ cells in the immunostaining images was performed by delineating 12 adjacent squares with a side length of 500 μm for the normal pancreas staining and 24 squares with a side length of 400 μm for the PDAC pancreas immunostaining. The number of positive cells for CD10 or GFAP, as well as cells that expressed both proteins, was computed using ImageJ software. CD68^+^ cells from tiled images were computed using ImageJ software and plotted versus the covered surface area. The quantification of microlesions in liver tissue from KPC and KPC;TRPV4-KO mice was conducted by identifying the presence of Ki67^+^ cells alongside CK19^+^ cells. These cells can form structures resembling ducts near blood vessels or exist as a mass. A mass containing at least 10 such cells was considered a single lesion.

### Statistics.

Comparisons of 2 values were analyzed by 2-tailed Student’s *t* test (GraphPad Prizm 10.1.0). Multiple comparisons were made using 1-way ANOVA with Dunnett’s post hoc test. Mouse survival statistics were analyzed using log-rank (Mantel-Cox) test. Results are expressed as the mean ± SEM. *P* < 0.05 was considered significant.

### Study approval.

Experimental protocols and all studies were performed with approval from the IACUC and IRB of Duke University (protocol no. Pro00035974).

### Data availability.

All the data values presented in figures of the main manuscript and supplemental material are provided in Supporting Data Values. RNA-Seq data are available through accession no. GSE296050.

## Author contributions

RAL, SMS, and JMJR designed the experiments. JMJR and SMS performed most of the experiments, analyzed the data, and contributed equally. RAL, JMJR, and SMS wrote the manuscript. NM and BB performed experiments. RAL supervised the project and provided funding for the project. All authors reviewed the manuscript.

## Funding support

This work is the result of NIH funding, in whole or in part, and is subject to the NIH Public Access Policy. Through acceptance of this federal funding, the NIH has been given the right to make the work publicly available in PubMed Central.

- Department of Veterans Affairs Merit Review Award I01BX006301
- NIH grants R01 DK120555 and R01 DK1244474

## Supplementary Material

Supplemental data

Supporting data values

## Version 1 10/16/2025

In-Press Preview

## Version 2 12/08/2025

Electronic publication
