# Supplementary material for: Loss of TRPV4 reduces pancreatic cancer growth and metastasis
Source: JCI Insight. 2025 Oct 16;10(23):e196280. doi: 10.1172/jci.insight.196280 (PMC12890521; doi:10.1172/jci.insight.196280)
Supplement: Supplemental data [file jciinsight-10-196280-s107.pdf]

SUPPLEMENTAL FIGURE LEGENDS

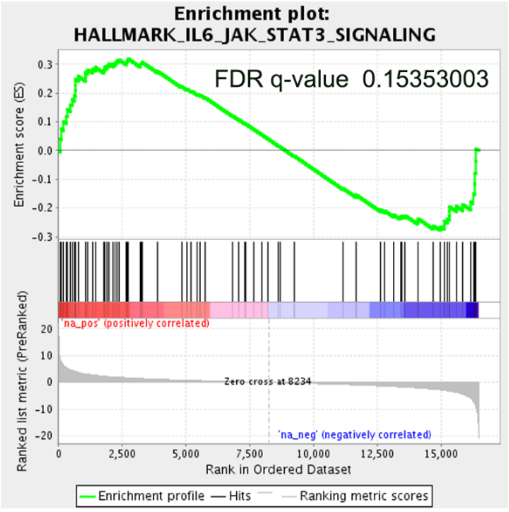

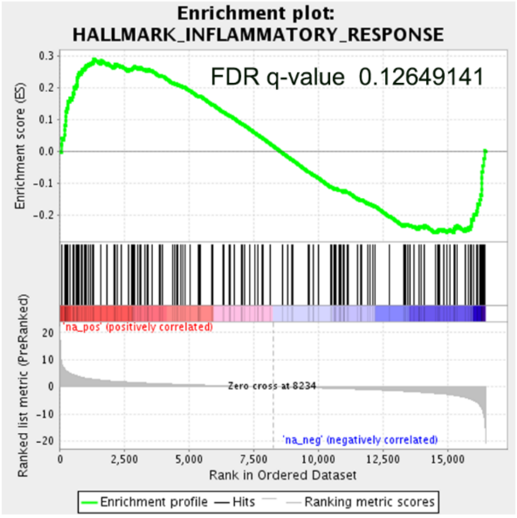

Supplemental Figure 1: Enrichment plots from a Gene Set Enrichment Analysis (GSEA) of PSCs that were induced with the PIEZO1 agonist Yoda1. GSEA was performed on mouse PSCs that were either untreated or treated with Yoda1 at a concentration of 25 μM for 72 hours. Hallmark gene sets indicate that IL-6_ JAK_STAT3, and Inflammatory Response genes were enriched in cells treated with Yoda1.

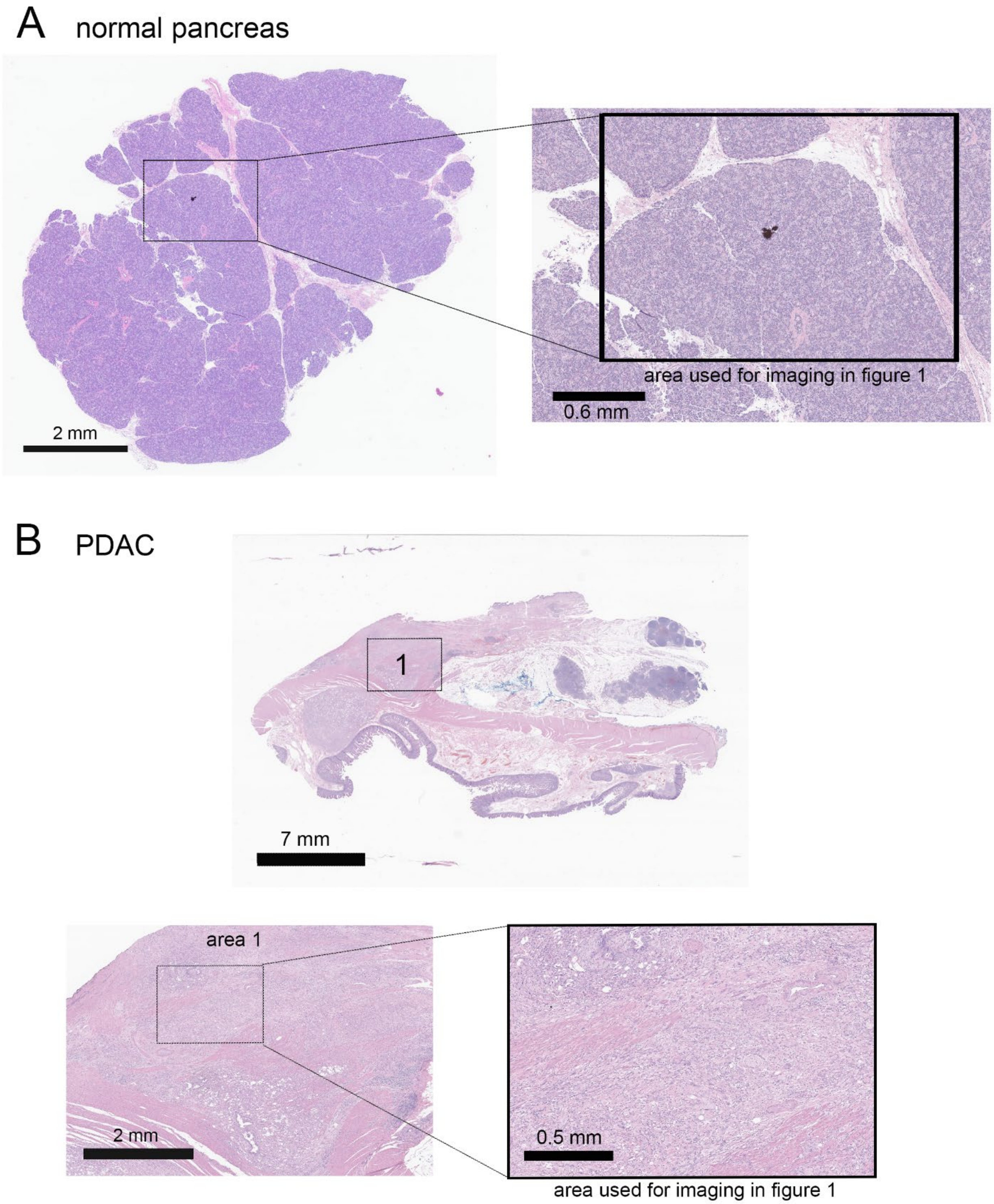

Supplemental Figure 2: H&E staining of normal human pancreata and PDAC samples. Panel (**A**) displays the H&E staining of a normal human pancreas, while panel (**B**) shows the staining of human PDAC tissue. Images were captured using a Leica Aperio GT 450 scanner with a 40x objective and were analyzed using Leica Aperio ImageScope software. The black rectangles indicate the area corresponding to the immunostaining presented in Figure 1D.

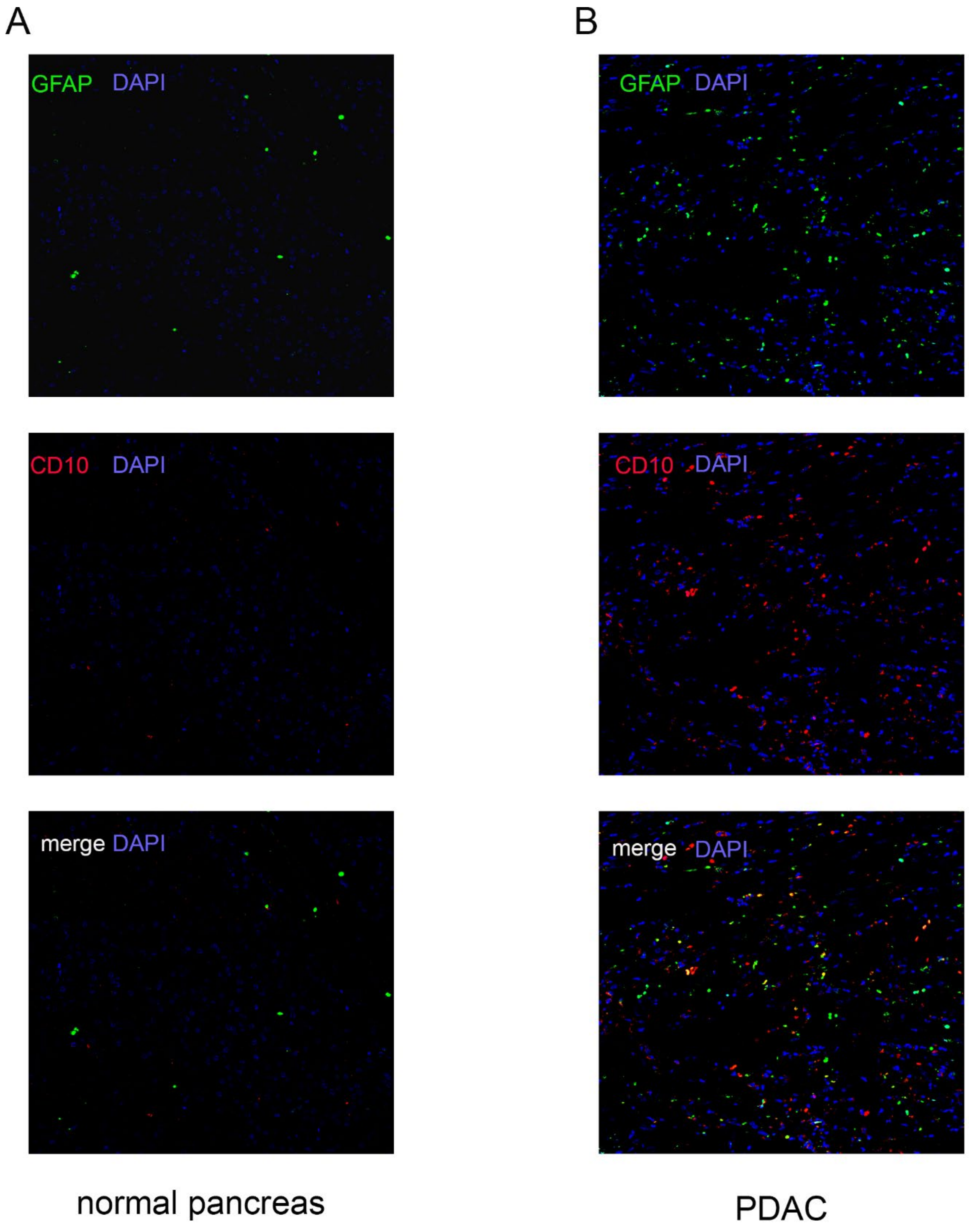

Supplemental Figure 3: Immunostaining of normal and PDAC human pancreas tissue sections using antisera specific for CD10 and GFAP. The images shown are representative of those presented in Figure 1D, which were used for quantifying cell types.

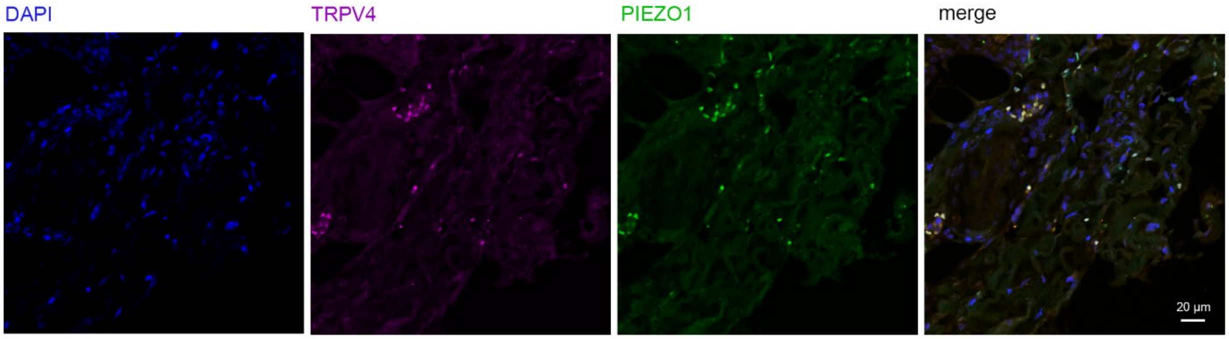

Supplemental Figure 4: Immunostaining of human PDAC tissue sections with PIEZO1 and TRPV4 antibodies. Formalin-fixed PDAC tissue was stained with TRPV4-ATTO-Fluor-550 antibody at a dilution of 1:100 (magenta) or with PIEZO1 antibody at a dilution of 1:100 (green).

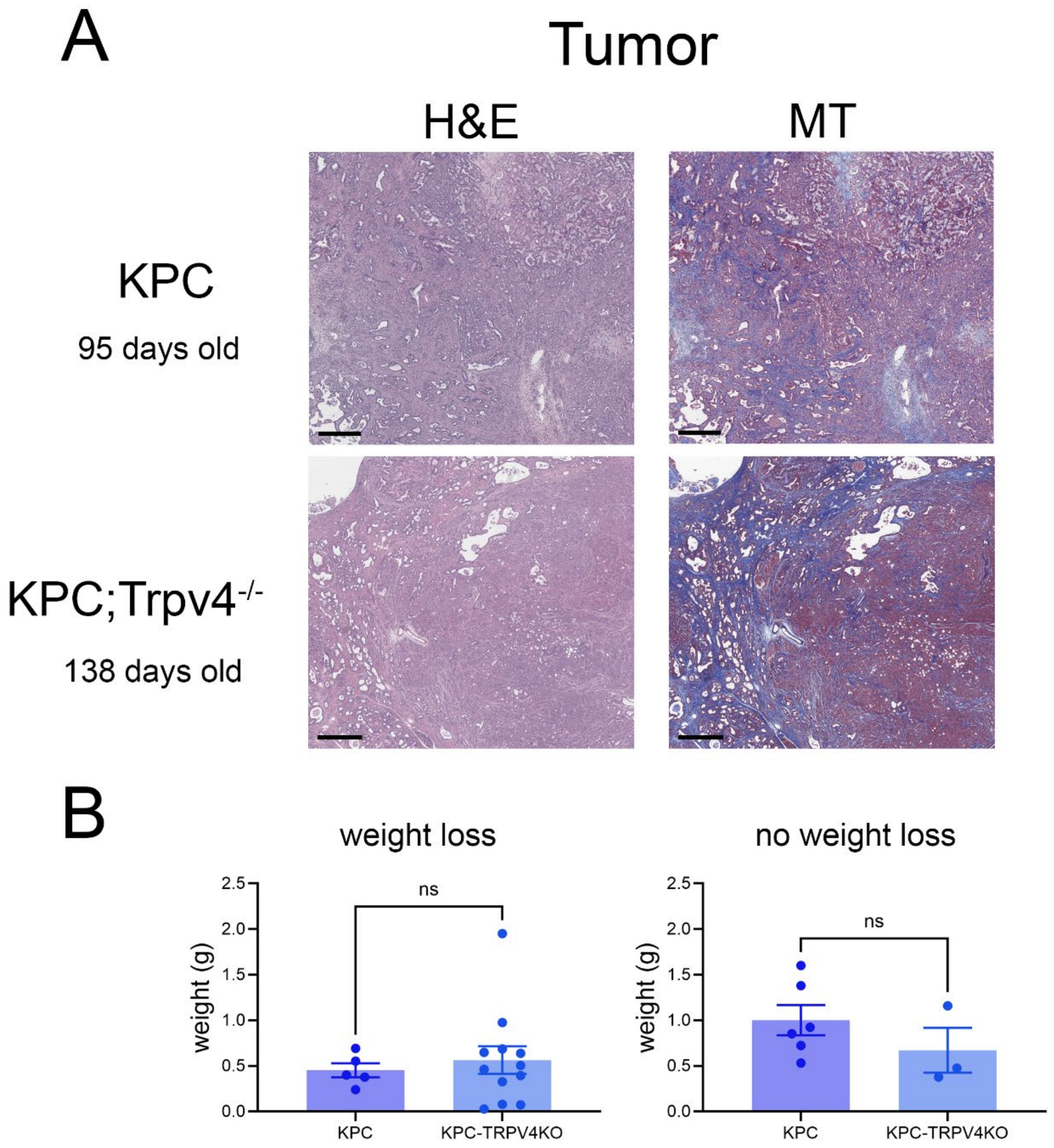

Supplemental Figure 5: Histology and Tumor Weight in KPC and KPC;TRPV4 KO Mice. (**A**) Histological analysis was performed using Hematoxylin and Eosin (H&E) staining and Masson's Trichrome (MT) staining on primary tumors from KPC GEMM and KPC;TRPV4 KO mice. Images were captured with a Leica Aperio GT 450 scanner using a 40x objective lens and analyzed with Aperio ImageScope software. Scale bar equals 1 mm. (**B**) The weight of primary tumors from non-surviving mice is shown. Left panel: mice that were euthanized due to significant weight loss; right panel: mice that did not exhibit weight loss but showed signs of distress (humane endpoint). Statistical analysis was conducted using Student's t-test and results are presented as mean ± SEM.
